# Women waste pickers’ perceptions of nursing care and environmentally sustainable practices: action research

**DOI:** 10.1590/1980-220X-REEUSP-2025-0227en

**Published:** 2026-05-11

**Authors:** Roseléia Regina Halmenschlager, Rosiane Aparecida Copetti Macht, Livia Brum de Brum, Alacoque Lorenzini Erdmann, Andreas Büscher, Dirce Stein Backes

**Affiliations:** 1 Universidade Franciscana, Programa de Pós-Graduação em Materno-Infantil, Santa Maria, RS, Brazil.; 2 Universidade Franciscana, Santa Maria, RS, Brazil.; 3 Universidade Federal de Santa Catarina, Programa de Pós-Graduação de Enfermagem, Florianópolis, SC, Brazil.; 4 University of Applied Sciences Osnabrück, Osnabrück, Germany.

**Keywords:** Nursing Care, Community Health Nursing, Sustainable Development, Environmental Health.

## Abstract

**Objective:**

To understand the perceptions of women waste pickers regarding nursing care in the promotion of environmentally sustainable practices, from the perspective of social entrepreneurship.

**Method:**

An action research study conducted between August 2024 and March 2025 with 26 women from a waste pickers’ association located in southern Brazil, focusing on the promotion of environmentally sustainable practices. This study focused solely on the investigative phase, during which data were collected through individual interviews and examined using reflexive thematic analysis.

**Results:**

The women identified nursing care as essential to the organization and safety of the workplace. Their perceptions suggest that the presence of a nurse in the association’s daily activities fostered recognition of the social value of their work and boosted their self-esteem. These findings reinforce the role of nursing care as a form of social entrepreneurship, intrinsically linked to environmental health.

**Conclusion:**

The perspectives of women waste pickers reveal that conceiving of nursing care as a driver of sustainability requires a break from the conventional model. From the perspective of social entrepreneurship, care emerges as a systemic and relational phenomenon, in which the nurse serves as the essential link between human health and planetary integrity.

## INTRODUCTION

Nursing plays a leading role in promoting eco-friendly initiatives to achieve the Sustainable Development Goals^(1,2)^. Studies highlight the interconnection between human health and ecosystem integrity, aiming to move beyond welfare-based approaches and promote sustainable practices^(3,4,5)^. In this context, nurses play a strategic role in environmental education and the mitigation of occupational risks^(6,7)^.

From the perspective of social entrepreneurship, this care focuses on collaborative leadership within vulnerable communities. By working at the intersection of life and the environment, nurses empower marginalized groups, transforming social challenges into opportunities for collective development^(8,9)^. Thus, care emerges as a systemic and entrepreneurial phenomenon, essential to human dignity and the sustainability of life in both its individual and collective dimensions^(10,11)^.

However, there remains a scientific gap regarding the integration of care into the daily practices of marginalized communities in contexts of socio-environmental vulnerability, specifically in the work context of women waste pickers. Previous studies underscore the urgency of moving beyond theoretical abstractions to investigate the potential of care to promote autonomy, dignity, and visibility for these traditionally marginalized groups^(6,12)^. From this perspective, nurses emerge as strategic agents, capable of integrating the preservation of life with local sustainable development^(13)^.

Based on the above, the following research question arises: What are the perceptions of women recyclers regarding nursing care in the promotion of environmentally sustainable practices? The objective is, therefore, to understand the perceptions of women recyclers regarding nursing care in the promotion of environmentally sustainable practices, from the perspective of social entrepreneurship.

## METHOD

### Study Design

This is an action research study^(14)^ conducted at a recycling association in southern Brazil, as part of the “Social Entrepreneurship in Nursing” project. The structure of this report followed the Consolidated Criteria for Reporting Qualitative Research (COREQ)^(15)^.

### Study Setting

The study population consisted of 32 workers, of whom 26 women made up the final sample (with no refusals). The inclusion criteria were: being female and having participated in previous interventions on sustainable practices (use of PPE, waste management, and self-care). Those on leave from work during data collection were excluded. The interventions took place weekly, following a schedule previously agreed upon with the participants, based on active methodologies grounded in social entrepreneurship.

### Data Collection and Organization

Data were collected between August 2024 and March 2025 through individual interviews, with an average duration of 20 minutes, on days, at times, and in a location (private space) previously agreed upon with each participant. The interviews were conducted by a researcher with a Ph.D. and expertise in qualitative research who had previously participated in the systematic interventions. The questions that guided the interviews were: Tell me about your work here at the Recycling Association. What do you understand by ecologically sustainable care? What is the relationship between ecological care and your well-being? What activities do you consider important that can promote well-being and ecologically sustainable practices? The data collection was terminated based on theoretical saturation, which was determined when the accounts ceased to provide new elements for the density of the codes and the further exploration of emerging themes^(16)^.

The researcher exercised reflexivity on an ongoing basis, beginning with her prior immersion in the field, which fostered a bond of trust and facilitated participant involvement in data collection. A field journal was used to distinguish the researcher’s impressions from the facts being narrated, thereby ensuring methodological rigor and self-criticism. The interpretations were presented to the participants at a specific meeting for validation and the addition of elements; this meeting was also audio-recorded.

### Data Analysis

Reflexive Thematic Analysis^(17)^ was used, following six phases: familiarization, code generation, theme identification, review, definition, and report production. The ATLAS.ti software (v.8.0) assisted in organizing the data. Two researchers performed independent coding (39 initial codes), which were refined by consensus with a third researcher, resulting in 37 final codes organized into three themes and nine subthemes. Independent coding by two researchers and refinement by consensus aimed to mitigate purely subjective interpretations, ensuring that the nuclei of meaning were anchored in empirical reality. The research dataset will be available from the corresponding author and accessible upon reasonable request.

### Ethical Considerations

Throughout the research process, the recommendations of Resolution No. 466/2012 of the National Health Council were followed. The research project was approved by the National Ethics and Research Committee under Opinion No. 5.615.579/2022. Participation was voluntary, subject to the signing of the Informed Consent Form and the guarantee of withdrawal at any time. To maintain anonymity, participants’ statements were identified throughout the text with the letter ‘W’ for Woman, followed by a number corresponding to the order of the statements: W1...W26. The data will be kept in the custody of the principal investigator for five years.

## RESULTS

The 26 women were between the ages of 19 and 64, with an average of four to eight children. Their workday consists of eight hours, with a monthly income ranging from 600.00 to 1,100.00 reais.

Data analysis yielded three central themes: Nursing Care versus Ecological Care; An environment that fosters togetherness and promotes well-being; Strategies that enhance well-being and ecological care. The detailed structure of themes, subthemes, and examples of data units is summarized in Table 1, followed by an analytical description of each thematic axis.

**Table 1:** Topics, subtopics, and record units.

Key themes	Subthemes	Recording units
Nursing Care versus Ecological Care	The identity of the environmental nurse. The inseparable link between human and planetary health. A systemic and holistic view of care.	“*We are Environmental Nurses”; “Everything is care, or we are not caring”; “Ecological care is linked to disease*.”
An inclusive environment that promotes well-being	The workplace as a refuge and source of support. Health as a broad and relational concept. The environment as a subject, not an object.	“*Here we talk and laugh”; “Health is connected to the environment”; “We can’t treat the environment as an object” “My well-being directly impacts the well-being of others”; “Health is related to cleanliness, the surroundings, and interpersonal relationships*.”
Strategies to enhance well-being and ecological care	Prioritizing mental and emotional health. Challenges of overload and an exhausting routine. Sustainability in workplace relationships.	“*I work best when I can relax my mind and body”; “My days are hectic—I go to bed late and get up early”; “Teamwork really improves our work*.”

### Nursing Care vs. Ecological Care

The participants identify a direct link between their recycling activities and the protection of public health. By calling themselves “Environmental Nurses,” these women redefine manual labor as a strategic act of caring for life and sustainability. The accounts demonstrate that this perception elevates the importance of waste sorting, transforming it into an environmental protection practice that benefits the community.

*Our care here is very important. We take care of the environment; we help keep the city clean and well-maintained. We are Environmental Nurses.* (W1)

*We care for the life of the planet, for everything, everything that exists. All things are part of the planet and need to be cared for. And who needs to care for them? All of us. Here we always remember and say that we are Environmental Nurses.* (W5)

An analysis of the participants’ statements reveals the inseparable link between the clinical and ecological dimensions of care. For the participants, the practice of nursing and recycling work converge on the common goal of ensuring environmental conditions conducive to life. This integration validates the understanding that human health depends on the integrity of ecosystems, which motivates the workers to recognize their role in maintaining planetary balance and preventing health hazards.

*You can’t separate one thing from another. Either we take care of everything, or we don’t take care of anything. A nurse cares for the patient, but the patient depends on the air they breathe; they depend on the climate, the water, cleanliness, and the environment.* (W10)

*We need care, and so everything needs care. And if we don’t care, what happens? More diseases! What we do here is take care of the health of the environment.* (W14)

The narratives emphasize that the health-disease process is influenced by climatic and sanitary conditions, as well as the preservation of natural resources. By pointing out that health care depends on air and water quality, the women demonstrate an awareness of socio-environmental determinants. This perception suggests that care must encompass the environment as a central element for sustaining life, broadening the perspective on traditional therapeutic practices.

*Health is linked to the air we breathe and the water we drink. We can’t separate our work from health care.* (W19)

The data reveal that waste sorters view nursing care as an entrepreneurial social practice. The workers’ identification with the figure of the “Environmental Nurse” makes it clear that the promotion of sustainable practices is understood as an extension of health care, in which waste sorting becomes the operational tool for ensuring human dignity and the vitality of the ecosystem.

### A Welcoming Atmosphere that Promotes well-being

For the participants, well-being is a phenomenon linked to the quality of the environment and the establishment of mutually supportive interpersonal interactions. From this perspective, the workplace at the association goes beyond its productive function to become a space of emotional support and security, acting as a protective factor that helps in coping with daily challenges. Despite structural difficulties, the workers value the camaraderie among colleagues as a central element for maintaining mental health and job satisfaction.

*I feel good working here at the Association. Sure, it’s tiring work that isn’t very well valued. But I feel really good here, because at home I don’t have the space or the right atmosphere. When we come to work, we have our coworkers to talk to, so to speak.* (W3)

The data reveal that the workplace is characterized as a space for subjective exchanges, where dialogue and mutual support among women help mitigate the strain of an exhausting routine. This perspective broadens the concept of health beyond the mere absence of illness, integrating it with the quality of relationships and the organization of the work environment. The participants associate workplace cleanliness and group harmony as vital components that directly impact morale and productivity.

*At work, we always feel good and supported. We’re always chatting and laughing. So that’s what care is all about—it’s indescribable.* (W5)

*Health is linked to cleanliness, the environment, and interpersonal relationships. Working in a clean place with close-knit colleagues makes all the difference in helping us get through the day.* (W12)

The accounts suggest that maintaining a safe work environment is viewed not merely as a technical standard, but as a prerequisite for the dignity of female workers. By treating the environment as a factor that influences emotional and physical well-being, women emphasize that maintaining an organized and welcoming space is a strategy for collective care. This attention to the environment is reflected in daily cooperation, where care for others and for the space merge to ensure the continuity of activities.

*We can’t treat the environment like an object… We take care of the warehouse because the warehouse takes care of us. If everything is dirty and messy, we all get sick together.* (W18)

The participants’ statements show that care practices, such as rest and active listening, are essential for sustaining resilience in the recycling environment. For the participants, collective well-being stems from a culture of care that harmonizes individual needs with the organization of the shared space. By recognizing the association as a space of refuge and support, the women validate the premise of social entrepreneurship that transforming the environmental reality depends on a solid relational foundation. Thus, the promotion of sustainable practices is enhanced when the nurse acts as a facilitator of an environment that integrates physical, emotional, and social health.

### Strategies to Promote well-being and Environmental Stewardship

The participants identify mental health—especially rest—as key strategies for maintaining productivity and quality of life. Their accounts show that, in the face of a physically demanding routine, the ability to relax the mind and body is seen as essential for performing work tasks effectively. For women, nursing care manifests itself in encouraging restorative breaks and providing guidance that helps balance work demands with personal needs, as follows:

*The most important thing is mental and emotional health, not so much physical health, which we manage to get by with. Everyone gets tired, but I think the most important thing is the mind. I need to feel good about myself and others. This is related to the environment, the atmosphere, and the air I breathe.* (W13)

*I think there should be activities focused on the mind, something that helps relax the mind and body. Sometimes there’s just too much going on in your head, right? The atmosphere gets heavy, exhausting, and mistakes happen.* (W16)

The data reveal that cooperation and teamwork serve as mechanisms for sustainability in workplace relationships. The solidarity among the recyclers is identified as a strategy that facilitates the flow of activities and reduces the impact of physical strain. This observation highlights that ecological care, to be effective, depends on a collective support structure where mutual aid among colleagues optimizes material sorting and fosters a more harmonious environment, as expressed below:

*Teamwork really improves our work. When we help each other, the load feels lighter and we’re able to get everything sorted out properly.* (W20)

*We help each other a lot here. If one of us is tired, the other steps in to help. That makes the day go by more smoothly, and we take better care of things.* (W24)

The statements indicate that the adoption of environmentally sustainable practices, such as the proper use of equipment and rigorous waste separation, is contingent upon the emotional well-being of the workers. By linking their state of mind to the quality of their work, the women emphasize that efficiency in recycling is not merely a technical matter but depends on motivational and relational factors. This understanding suggests that nurses, by promoting mental health, indirectly strengthen environmental preservation.

*To take care of the environment, we have to feel good about ourselves. If I’m happy and well-rested, I take much better care of everything that comes my way.* (W21)

In short, the results demonstrate that the promotion of environmentally sustainable practices occurs through care strategies that value the human and collective dimensions of work. From the perspective of social entrepreneurship, the results show that nurses enhance planetary sustainability by intervening in the mental health and work organization of waste pickers. Thus, strengthening these women’s autonomy and emotional well-being establishes itself as the operational foundation for sustaining life and environmental balance.

## DISCUSSION

The results reveal that, in the participants’ perception, nursing care and ecological care are inseparable, forming a network of vital interdependence. By using the symbolic metaphor of “Environmental Nurses,” the waste sorters affirm the power of care beyond the phenomenon of illness and link waste sorting to the promotion of public health. This finding aligns with contemporary literature that positions nursing as a central category in the defense of planetary health, reviving the profession’s historical legacy regarding environmental health as a principle of healing^(18,19,20)^.

The atmosphere of the recycling association was identified as a critical determinant of health, as it serves as a space for emotional support that transcends its productive function. Well-being, in this context, is described by the women as a relational phenomenon, where the cleanliness of the surroundings and the harmony of interpersonal bonds act as protective factors against the wear and tear of an exhausting routine. Studies in occupational health reinforce that balance in work environments is a dynamic system, emphasizing that the quality of relationships and the organization of space are fundamental to workers’ dignity and mental health^(21,22)^.

The connection between the work of waste pickers and nursing care demonstrates that the impact of waste management on mitigating environmental damage is a shared ethical commitment. The women’s perception that “the patient depends on the air and the environment” underscores the urgency of moving beyond the traditional biomedical model by integrating socio-environmental determinants into daily care practices. Research reinforces this perspective by demonstrating that this expansion of the professional scope institutionalizes sustainability as a cornerstone of global health promotion^(21,22,23,24)^.

From the perspective of social entrepreneurship, the leading role played by waste pickers reveals that care can be a transformative force that fosters autonomy in vulnerable communities. By turning social challenges into opportunities for collective development, the participants demonstrate that entrepreneurial nursing care is not merely an ancillary tool, but a dimension that gives tangible meaning to community work. This approach enables the creation of creative solutions that harmonize the effectiveness of care with environmental responsibility, raising the visibility of traditionally marginalized groups^(25,26,27,28,29)^.

The convergence between the knowledge of waste pickers and nursing care demonstrates that the act of caring is directly linked to environmental preservation and human health. The findings indicate that care, when applied to the context of recycling, promotes health protection and improved living conditions. The study supports this view by reinforcing the role of nurses in risk management and the promotion of sustainable work, in order to safeguard the continuity of life in all its dimensions^(30)^.

From the perspective of social entrepreneurship, the leading role played by waste pickers reveals that nursing care acts as a catalyst for autonomy by integrating health guidance into their daily work routine^(11,12)^. The conversion of social challenges into opportunities for development is evidenced by the participants’ ability to self-manage risks and value collective work, giving a practical and tangible meaning to nursing practice in the community.

The limitations of this study relate to the specificity of the sample, which consists of women belonging to a single waste pickers’ association, thereby limiting the findings to this particular socio-occupational context. Due to its qualitative nature, the results do not aim for generalization, but rather for an in-depth analysis of experiences and motivations influenced by gender dynamics and unique working conditions.

The contributions of this study to the advancement of nursing science are linked to the urgency of integrating the ecological dimension as a fundamental principle of public health. The results provide a basis for the development and strengthening of public policies that promote shared responsibility in the ethical management of solid waste and the recognition of nurses as strategic agents at the intersection of human life and planetary balance.

## CONCLUSION

The waste pickers’ perceptions reveal that nursing care, by strengthening the autonomy of women waste pickers, promotes occupational safety, workplace organization, and emotional support. The presence of a nurse in the association’s daily activities serves as a public health surveillance intervention, transforming material sorting into a conscious practice of environmental preservation. The study highlights that integrating women’s knowledge with technical health guidelines enhances the adoption of sustainable practices and the appreciation of their professional identity.

It is concluded that nursing care, from the perspective of social entrepreneurship, transcends the clinical model by directly intervening in socio-environmental determinants and workplace dignity. Finally, it is suggested that future research on recyclers include other associations and male workers, in order to ensure greater representativeness and diversity, based on the particularities of each group, such as organization, working conditions, internal dynamics, and others

## Data Availability

The entire dataset supporting the results of this study is available upon request to the corresponding author.
