# Supplementary material for: Women waste pickers’ perceptions of nursing care and environmentally sustainable practices: action research
Source: Rev Esc Enferm USP. 2026 May 11;60:e20250227. doi: 10.1590/1980-220X-REEUSP-2025-0227en (PMC13160412; doi:10.1590/1980-220X-REEUSP-2025-0227en)
Supplement: Translation

# Translation: Percepções de mulheres recicladoras sobre o cuidado de enfermagem e práticas ecologicamente sustentáveis: pesquisa-ação

**DOI:** 10.1590/1980-220X-REEUSP-2025-0227pt

**Authors:** Roseléia Regina Halmenschlager, Rosiane Aparecida Copetti Macht, Livia Brum de Brum, Alacoque Lorenzini Erdmann, Andreas Büscher, Dirce Stein Backes

**Affiliations:** 1 Universidade Franciscana, Programa de Pós-Graduação em Materno-Infantil, Santa Maria, RS, Brazil.; 2 Universidade Franciscana, Santa Maria, RS, Brasil.; 3 Universidade Federal de Santa Catarina, Programa de Pós-Graduação de Enfermagem, Florianópolis, SC, Brasil.; 4 University of Applied Sciences Osnabrück, Osnabrück, Germany.

**Keywords:** Cuidado de Enfermagem, Enfermagem em Saúde Comunitária, Desenvolvimento Sustentável, Meio Ambiente e Saúde Pública.

## Abstract

**Objetivo:**

Compreender as percepções de mulheres recicladoras sobre o cuidado de Enfermagem na promoção de práticas ecologicamente sustentáveis, na perspectiva do empreendedorismo social.

**Método:**

Pesquisa-ação desenvolvida, entre agosto/2024 e março/2025, com 26 mulheres de uma Associação de recicladores localizada no sul do Brasil, a partir da promoção de práticas ecologicamente sustentáveis. Considerou-se, neste estudo, apenas a fase investigativa, cujos dados foram coletados por meio de entrevistas individuais e examinados à luz da análise temática do tipo *Reflexive*.

**Resultados:**

As mulheres identificaram o cuidado de Enfermagem como suporte essencial à organização e segurança do ambiente de trabalho. As percepções indicam que a presença do enfermeiro no cotidiano da associação promoveu o reconhecimento do valor social de seu trabalho e o fortalecimento da autoestima. Tais achados consolidam o cuidado como prática de empreendedorismo social, intrinsecamente associada à saúde ambiental.

**Conclusão:**

As percepções de mulheres recicladoras revelam que conceber o cuidado de Enfermagem como promotor de sustentabilidade requer a ruptura com o modelo convencional. Sob o prisma do empreendedorismo social, o cuidar consolida-se como um fenômeno sistêmico e relacional, no qual o enfermeiro é o elo essencial entre a saúde humana e a integridade planetária.

## INTRODUÇÃO

A Enfermagem desempenha papel protagonista na promoção de ações eco-orientadas para o alcance dos Objetivos de Desenvolvimento Sustentável^(1,2)^. Estudos enfatizam a interconexão entre saúde humana e integridade ecossistêmica, visando transcender abordagens assistencialistas para consolidar condutas sustentáveis^(3,4,5)^. Nesse cenário, o Enfermeiro assume função estratégica na educação ambiental e na mitigação de riscos ocupacionais^(6,7)^.

Sob a ótica do empreendedorismo social, esse cuidado converge para o protagonismo colaborativo junto a comunidades vulneráveis. Ao atuar na confluência entre a vida e o meio ambiente, o Enfermeiro potencializa o saber de grupos marginalizados, convertendo desafios sociais em oportunidades de desenvolvimento coletivo^(8,9)^. Assim, o cuidado afirma-se como fenômeno sistêmico e empreendedor, essencial à dignidade humana e à sustentabilidade da vida em sua dimensão individual e coletiva^(10,11)^.

Contudo, persiste uma lacuna científica sobre a articulação do cuidado à práxis de comunidades periféricas em contextos de vulnerabilidade socioambiental, mais especificamente no context laboral de mulheres recicladoras. Estudos previamente realizados ratificam a urgência de transpor abstrações teóricas para investigar o potencial do cuidado na promoção da autonomia, dignidade e visibilidade a esses grupos tradicionalmente marginalizados^(6,12)^. Nessa perspectiva, o Enfermeiro emerge como agente estratégico, capaz de integrar a preservação da vida ao desenvolvimento sustentável local^(13)^.

Tem-se, com base no exposto, a seguinte questão pesquisa: quais as percepções de mulheres recicladoras sobre o cuidado de Enfermagem na promoção de práticas ecologicamente sustentáveis? Objetiva-se, para tanto, compreender as percepções de mulheres recicladoras sobre o cuidado de Enfermagem na promoção de práticas ecologicamente sustentáveis, na perspectiva do empreendedorismo social.

## MÉTODO

### Desenho do Estudo

Trata-se de uma pesquisa-ação^(14)^ realizada em uma associação de reciclagem no Sul do Brasil, vinculada ao projeto “Empreendedorismo Social da Enfermagem”. A estrutura do relato seguiu os critérios do Consolidated Criteria for Reporting Qualitative Research (COREQ)^(15)^.

### Cenário de Estudo

A população foi composta por 32 trabalhadores, dos quais 26 mulheres compuseram a amostra final (sem recusas). Os critérios de inclusão foram: ser mulher e ter participado das intervenções prévias sobre práticas sustentáveis (uso de EPIs, gestão de resíduos e autocuidado). Excluíram-se aquelas em afastamento laboral durante a coleta. As intervenções ocorreram semanalmente, a partir de um cronograma previamente acordado com os participantes, fundamentadas em metodologias ativas sustentadas no empreendedorismo social.

### Coleta e Organização dos Dados

Os dados foram coletados, entre agosto/2024 e março/2025, por meio de entrevistas individuais, com duração média de 20 minutos, em dias, horários e local (espaço privado) previamente acordados com cada uma das participantes. As entrevistas foram conduzidas por uma pesquisadora doutora com expertise qualitativa e que, previamente, havia participado das intervenções sistemáticas. As questões que nortearam as entrevistas foram: Fale-me sobre o seu trabalho aqui na Associação de Reciclagem. O que você entende por cuidado ecologicamente sustentável? Qual a relação entre o cuidado ecológico e o seu bem-estar? Quais atividades você considera importantes e que podem promover o bem-estar e práticas ecologicamente sustentáveis? O encerramento da coleta foi determinado pela saturação teórica, verificada quando os relatos deixaram de fornecer novos elementos para a densidade dos códigos e o aprofundamento dos temas emergentes^(16)^.

A reflexividade da pesquisadora foi exercida de modo contínuo, a partir de inserção prévia no campo, a qual possibilitou o vínculo de confiança e a participação na coleta de dados. Utilizou-se o diário de campo para separar as impressões da pesquisadora dos fatos narrados, de modo a garantir o rigor metodológico e a autocrítica. As interpretações foram apresentadas às participantes em encontro específico para validação e acréscimo de elementos, momento também audiogravado.

### Análise dos Dados

Utilizou-se a Análise Temática *Reflexive* ^(17)^, a partir das seis fases: familiarização, geração de códigos, busca por temas, revisão, definição e produção do relatório. O software ATLAS.ti (v.8.0) auxiliou na organização dos dados. Duas pesquisadoras realizaram a codificação independente (39 códigos iniciais), refinados em consenso com um terceiro pesquisador, resultando em 37 códigos finais organizados em três temas e nove subtemas. A codificação independente por duas pesquisadoras e o refinamento por consenso visaram mitigar interpretações puramente subjetivas, assegurando que os núcleos de sentido estivessem ancorados na realidade empírica. O conjunto de dados de pesquisa ficará disponível com o autor correspondente e acessáveis mediante solicitação razoável.

### Aspectos Éticos

Observou-se, ao longo do processo de pesquisa, as recomendações da Resolução n°. 466/2012 do Conselho Nacional de Saúde. O projeto de pesquisa foi aprovado pela Comissão Nacional de Ética e Pesquisa, sob o parecer 5.615.579/2022. A participação foi voluntária, mediante assinatura do Termo de Consentimento Livre e Esclarecido e garantia de desistência a qualquer momento. Para manter o anonimato, as falas das participantes foram identificadas, ao longo do texto, com a letra ‘M’, de Mulher, seguida por um algarismo correspondente à ordem das falas: M1...M26. Os dados serão mantidos sob guarda da pesquisadora principal por cinco anos.

## RESULTADOS

As 26 mulheres possuíam idade entre 19 e 64 anos, com média de quatro a oito filhos. A rotina laboral é de oito horas diárias, com rendimento mensal entre 600,00 e 1.100,00 reais.

A análise dos dados resultou em três temáticas centrais, quais sejam: Cuidado de Enfermagem *versus* Cuidado Ecológico; Ambiência agregadora e promotora de bem-estar; Estratégias potencializadoras de bem-estar e de cuidado ecológico. A estrutura detalhada de temas, subtemas e exemplos de unidades de registro está sintetizada na Tabela 1, seguida pela descrição analítica de cada eixo temático.

**Tabela 1:** Temas, subtemas e unidades de registro.

Temas centrais	Subtemas	Unidades de registro
Cuidado de Enfermagem versus Cuidado Ecológico	A identidade da Enfermeira do meio ambiente. Indissociabilidade entre saúde humana e planetária. Visão sistêmica e integral do cuidado.	“*Nós somos Enfermeiras do Meio Ambiente”; “Tudo é cuidado ou não cuidamos”; “O cuidado ecológico tem relação com as doenças*.
Ambiência agregadora e promotora de bem-estar	O espaço de trabalho como refúgio e apoio. Saúde como conceito ampliado e relacional. O meio ambiente como sujeito e não objeto.	“*Aqui conversamos e damos risadas”; “A saúde tem conexão com o meio ambiente”; “Não podemos tratar o ambiente como objeto”; “O meu bem-estar impacta diretamente no bem-estar da outra”; “A saúde está relacionada à limpeza, ao entorno e às relações interpessoais”*.
Estratégias potencializadoras de bem-estar e de cuidado ecológico	Priorização da saúde mental e emocional. Desafios da sobrecarga e da rotina exaustiva. Sustentabilidade nas relações de trabalho.	“*Eu trabalho melhor quando consigo relaxar a mente e o corpo”; “Os dias são corridos, durmo tarde e levanto cedo”; “O trabalho em equipe melhora muito o nosso trabalho*”.

### Cuidado De Enfermagem versus Cuidado Ecológico

As participantes identificam uma interconexão direta entre suas atividades de reciclagem e a preservação da saúde pública. Ao atribuírem a si o título de ‘Enfermeiras do Meio Ambiente’, as mulheres ressignificam o trabalho manual como uma ação estratégica de cuidado com a vida e com a sustentabilidade. Os relatos demonstram que essa percepção eleva a importância do trabalho de triagem de resíduos, transformando-o em uma prática de proteção ambiental que beneficia a coletividade.

*O nosso cuidado aqui é muito importante. Cuidamos do meio ambiente, ajudamos para manter a cidade limpa e bem cuidada. Nós somos Enfermeiras do Meio Ambiente*. (M1)

*Nós cuidamos da vida do Planeta, de tudo, tudo o que existe. Todas as coisas fazem parte do Planeta e precisam ser cuidadas. E, quem precisa cuidar? Todos nós. Aqui nós sempre lembramos e falamos que somos Enfermeiras do Meio Ambiente.* (M5)

A análise das falas revela a indissociabilidade entre as dimensões assistencial e ecológica do cuidado. Para as participantes, o exercício da Enfermagem e o trabalho de reciclagem convergem no objetivo comum de garantir condições ambientais favoráveis à vida. Essa integração valida a compreensão de que a saúde humana depende da integridade dos ecossistemas, o que motiva as trabalhadoras a reconhecerem seu papel na manutenção do equilíbrio planetário e na prevenção de agravos à saúde.

*Não dá para separar uma coisa da outra. Tudo é cuidado ou não cuidamos. Um Enfermeiro cuida do paciente, mas o paciente depende do ar que respira, ele depende do clima, da água, da limpeza, do meio ambiente.* (M10)

*Nós precisamos do cuidado e assim todas as coisas precisam de cuidado. E, se nós não cuidamos, o que acontece? Mais doenças! O que nós fazemos aqui é cuidar da saúde do meio ambiente.* (M14)

As narrativas reiteram que o processo saúde-doença é influenciado pelas condições climáticas, sanitárias e pela preservação dos recursos naturais. Ao pontuarem que a assistência à saúde depende da qualidade do ar e da água, as mulheres demonstram consciência sobre os determinantes socioambientais. Essa percepção indica que o cuidado deve abranger o ambiente como elemento central para a manutenção da vida, ampliando a visão sobre as práticas terapêuticas tradicionais.

*A saúde está relacionada com o ar que respiramos, a água que tomamos. Não dá para separar o nosso trabalho com o cuidado da saúde.* (M19)

Os dados revelam que as recicladoras percebem o cuidado de Enfermagem como prática social empreendedora. A identificação das trabalhadoras com a figura da ‘Enfermeira do Meio Ambiente’ explicita que a promoção de práticas sustentáveis é compreendida como uma extensão do cuidado à saúde, onde a triagem de resíduos se torna a ferramenta operacional para garantir a dignidade humana e a vitalidade do ecossistema.

### Ambiência Agregadora e Promotora de Bem-Estar

Para as participantes, o bem-estar configura-se como um fenômeno vinculado à qualidade da ambiência e ao estabelecimento de interações interpessoais de apoio mútuo. Sob essa ótica, o cenário laboral na associação transcende a função produtiva para tornar-se um espaço de suporte emocional e segurança, atuando como um fator de proteção que auxilia no enfrentamento de desafios cotidianos. A despeito das dificuldades estruturais, as trabalhadoras valorizam a convivência entre as colegas como um elemento central para a manutenção da saúde mental e da satisfação no trabalho.

*Eu me sinto bem trabalhando aqui na Associação. Claro é um serviço cansativo e pouco valorizado. Mas, eu me sinto muito bem aqui, porque em casa não tenho espaço e ambiente. Quando a gente vem para o trabalho, a gente tem as colegas para conversar, como eu vou dizer.* (M3)

Os dados revelam que o cenário laboral se caracteriza como um território de trocas subjetivas, onde o diálogo e o acolhimento entre as mulheres minimizam o desgaste da rotina exaustiva. Essa percepção amplia o conceito de saúde para além da ausência de doenças, integrando-o à qualidade das relações e à organização do entorno. As participantes associam a limpeza do local de trabalho e a harmonia do grupo como componentes vitais que impactam diretamente no ânimo e na capacidade produtiva.

*No trabalho a gente se sente sempre bem e apoiada. A gente tá sempre conversando e dando risada. Então, este é o sentido do cuidado, não tem palavras*. (M5)

*A saúde está relacionada à limpeza, ao entorno e às relações interpessoais. Trabalhar em um lugar limpo e com as colegas unidas faz toda a diferença para a gente aguentar o dia.* (M12)

As narrativas indicam que a preservação do ambiente de trabalho não é vista apenas como uma norma técnica, mas como uma condição para a dignidade das trabalhadoras. Ao tratarem o meio ambiente como um elemento que influencia o estado emocional e físico, as mulheres reforçam que a manutenção de um espaço organizado e acolhedor é uma estratégia de cuidado coletivo. Esse zelo com a ambiência reflete-se na cooperação diária, onde o cuidado com o outro e com o espaço se fundem para garantir a continuidade das atividades.

*Não podemos tratar o ambiente como objeto... A gente cuida do galpão porque o galpão cuida da gente. Se está tudo sujo e bagunçado, a gente adoece junto.* (M18)

As falas demonstram que práticas de cuidado, como o descanso e a escuta ativa, são fundamentais para sustentar a resiliência no ambiente da reciclagem. Para as participantes, o bem-estar coletivo é fruto de uma cultura de cuidado que harmoniza as necessidades individuais com a organização do espaço compartilhado. Ao reconhecerem a associação como um espaço de refúgio e suporte, as mulheres validam a premissa do empreendedorismo social de que a transformação da realidade ambiental depende de uma base relacional sólida. Assim, a promoção de práticas sustentáveis é potencializada quando o Enfermeiro atua como facilitador de uma ambiência que integra saúde física, emocional e social.

### Estratégias Potencializadoras de Bem-Estar e de Cuidado Ecológico

As participantes identificam a saúde mental, especialmente o descanso, como estratégias fundamentais para a manutenção da produtividade e da qualidade de vida. Os relatos demonstram que, diante de uma rotina física exaustiva, a capacidade de relaxar a mente e o corpo é percebida como uma condição para o bom desempenho das atividades laborais. Para as mulheres, o cuidado de Enfermagem manifesta-se no estímulo a pausas restauradoras e em orientações que auxiliam no equilíbrio entre as demandas do trabalho e as necessidades pessoais, conforme segue:

*O principal é a saúde mental e emocional, não tanto o físico, que a gente vai levando. Cansar todo mundo cansa, mas eu acho que o principal é a cabeça, a mente. Eu me sentir bem comigo mesma e com os outros. Isso tem relação com o ambiente, com o clima, com o ar que eu respiro*. (M13)

*Eu acho que teria que ter atividades voltadas para a mente, alguma coisa que ajudasse a relaxar a mente e o corpo. Às vezes é muita coisa na cabeça, né. O ambiente fica pesado, cansativo, acontecem erros.* (M16)

Os dados revelam que a cooperação e o trabalho em equipe atuam como mecanismos de sustentabilidade nas relações de trabalho. A união entre as recicladoras é apontada como uma estratégia que facilita o fluxo das atividades e reduz o impacto da sobrecarga física. Essa percepção evidencia que o cuidado ecológico, para ser efetivo, depende de uma estrutura de suporte coletivo onde a ajuda mútua entre as colegas otimiza a triagem dos materiais e promove um ambiente mais harmonioso, conforme expresso, a seguir:

*O trabalho em equipe melhora muito o nosso trabalho. Quando uma ajuda a outra, o fardo fica mais leve e a gente consegue separar tudo direitinho.* (M20)

*A gente se ajuda muito aqui. Se uma está cansada, a outra dá uma força. Isso faz o dia passar melhor e a gente cuida melhor das coisas.* (M24)

As falas indicam que a adoção de práticas ecologicamente sustentáveis, como o uso correto de equipamentos e a separação rigorosa dos resíduos, está condicionada ao bem-estar emocional das trabalhadoras. Ao associarem o estado de espírito à qualidade da execução das tarefas, as mulheres reforçam que a eficiência na reciclagem não é apenas técnica, mas depende de condições motivacionais e relacionais. Esse entendimento sugere que o Enfermeiro, ao promover a saúde mental, fortalece indiretamente a preservação ambiente.

*Para cuidar do meio ambiente, a gente tem que estar bem com a gente mesma. Se eu estou feliz e descansada, eu cuido muito melhor de cada material que passa por mim*. (M21)

Os resultados demonstram, em suma, que a promoção de práticas ecologicamente sustentáveis ocorre por meio de estratégias de cuidado que valorizam a dimensão humana e coletiva do trabalho. Sob a ótica do empreendedorismo social, os resultados evidenciam que o Enfermeiro potencializa a sustentabilidade planetária ao intervir na saúde mental e na organização do trabalho das recicladoras. Assim, o fortalecimento da autonomia e do bem-estar emocional dessas mulheres consolida-se como a base operacional para a manutenção da vida e do equilíbrio ambiental.

## DISCUSSÃO

Os resultados revelam que, na percepção das participantes, o cuidado de Enfermagem e o cuidado ecológico são indissociáveis, compondo uma rede de interdependência vital. Ao utilizarem a metáfora simbólica de ‘Enfermeiras do Meio Ambiente’, as recicladoras validam a potência do cuidado para além do fenômeno doença e correlacionam a triagem de resíduos à promoção da saúde coletiva. Esse achado converge com a literatura contemporânea que posiciona a Enfermagem como categoria central na defesa da saúde planetária, resgatando o legado histórico da profissão sobre a salubridade ambiental como princípio da cura^(18,20)^.

A ambiência da associação de reciclagem foi identificada como um determinante crítico de saúde, ao figurar como um território de suporte emocional que transcende a função produtiva. O bem-estar, nesse contexto, é relatado pelas mulheres como um fenômeno relacional, onde a limpeza do entorno e a harmonia dos vínculos interpessoais atuam como fatores de proteção contra o desgaste da rotina exaustiva. Estudos em saúde ocupacional reforçam que o equilíbrio em ambientes laborais é um sistema dinâmico, ao enfatizarem que a qualidade das relações e a organização do espaço são fundamentais para a dignidade e a saúde mental do trabalhador^(21,22)^.

A articulação entre a prática das recicladoras e o cuidado de Enfermagem demonstra que o impacto da gestão de resíduos na mitigação de danos ambientais é um compromisso ético compartilhado. A percepção das mulheres de que “o paciente depende do ar e do meio ambiente” ratifica a urgência de superar o modelo biomédico tradicional, integrando os determinantes socioambientais ao cotidiano assistencial. Investigações reforçam esse pensar ao demonstrarem que essa expansão do escopo profissional institucionaliza a sustentabilidade como eixo estruturante da promoção da saúde global^(21,24)^.

Sob a perspectiva do empreendedorismo social, o protagonismo das recicladoras revela que o cuidado pode ser uma força transformadora e geradora de autonomia em comunidades vulneráveis. Ao converterem desafios sociais em oportunidades de desenvolvimento coletivo, as participantes demonstram que o cuidado empreendedor de Enfermagem não é apenas uma ferramenta acessória, mas uma dimensão que confere significado tangível ao trabalho comunitário. Essa lógica permite gerar soluções criativas que harmonizam a eficácia do cuidado com a responsabilidade ambiental, elevando a visibilidade de grupos tradicionalmente marginalizados^(25,29)^.

A convergência entre o saber das recicladoras e o cuidado de enfermagem demonstra que o ato de cuidar está diretamente ligado à preservação do ambiente e à saúde humana. Os achados indicam que o cuidado, quando aplicado ao contexto da reciclagem, promove a proteção da saúde e a melhoria das condições de vida. Estudo corrobora com esse pensar ao reforçar o papel do Enfermeiro na gestão de riscos e na valorização do trabalho sustentável, de modo a salvaguardar a perenidade da vida em sua multidimensionalidade^(30)^.

Sob a perspectiva do empreendedorismo social, o protagonismo das recicladoras revela que o cuidado de Enfermagem atua como ferramenta propulsora de autonomia ao integrar orientações de saúde à rotina produtiva^(11,12)^. A conversão de desafios sociais em oportunidades de desenvolvimento é evidenciada pela capacidade das participantes em autogerir riscos e valorizar o trabalho coletivo, conferindo um sentido prático e tangível à atuação da Enfermagem na comunidade.

As limitações deste estudo relacionam-se à especificidade do recorte amostral, composto por mulheres pertencentes a uma única associação de recicladores, o que circunscreve os achados a esse contexto socio-laboral particular. Devido à natureza qualitativa, os resultados não buscam a generalização, mas a profundidade analítica de experiências e motivações que são influenciadas por dinâmicas de gênero e condições de trabalho singulares.

As contribuições deste estudo para o avanço da ciência de Enfermagem estão associadas à premência de integrar a dimensão ecológica como princípio basilar da saúde pública. Os resultados oferecem subsídios para a indução e o fortalecimento de políticas públicas que estimulam a corresponsabilidade no manejo ético dos resíduos sólidos e na valorização do Enfermeiro como agente estratégico na intersecção entre vida humana e equilíbrio planetário.

## CONCLUSÃO

As percepções das recicladoras revelam que o cuidado de Enfermagem, ao fortalecer a autonomia das mulheres recicladoras, promove a segurança ocupacional, a organização do ambiente de trabalho e o suporte emocional. A presença do Enfermeiro no cotidiano da associação atua como uma intervenção de vigilância em saúde, convertendo a triagem de materiais em uma prática consciente de preservação ambiental. O estudo evidencia que a integração do saber das mulheres às orientações técnicas de saúde potencializa a adoção de práticas sustentáveis e a valorização de sua identidade profissional.

Conclui-se que o cuidado de Enfermagem, sob a ótica do empreendedorismo social, transcende o modelo clínico ao intervir diretamente nos determinantes socioambientais e na dignidade laboral. Sugere-se, enfim, que pesquisas futuras sobre recicladores contemplem outras associações e, também, trabalhadores do sexo masculino, a fim de assegurar maior representatividade e diversidade, a partir das particularidades de cada grupo, como organização, condições de trabalho, dinâmicas internas e outros.

## Data Availability

Todo o conjunto de dados que dá suporte aos resultados deste estudo está disponível mediante solicitação ao autor correspondente.

# Translation: Percepciones de mujeres recicladoras sobre la atención de enfermería y prácticas ecológicamente sostenibles: investigación-acción

**DOI:** 10.1590/1980-220X-REEUSP-2025-0227es

**Authors:** Roseléia Regina Halmenschlager, Rosiane Aparecida Copetti Macht, Livia Brum de Brum, Alacoque Lorenzini Erdmann, Andreas Büscher, Dirce Stein Backes

**Affiliations:** 1 Universidade Franciscana, Programa de Pós-Graduação em Materno-Infantil, Santa Maria, RS, Brazil.; 2 Universidade Franciscana, Santa Maria, RS, Brasil.; 3 Universidade Federal de Santa Catarina, Programa de Pós-Graduação de Enfermagem, Florianópolis, SC, Brasil.; 4 University of Applied Sciences Osnabrück, Osnabrück, Germany.

**Keywords:** Atención de Enfermería, Enfermería en Salud Comunitaria, Desarrollo Sostenible, Medio Ambiente y Salud Pública.

## Abstract

**Objetivo:**

Vislumbrar las percepciones de mujeres recicladoras sobre la atención de enfermería en la promoción de prácticas ecológicamente sostenibles, desde la perspectiva del emprendimiento social.

**Método:**

Se trata de una investigación-acción llevada a cabo entre agosto de 2024 y marzo de 2025 con 26 mujeres de una asociación de recicladores del sur de Brasil, centrada en la promoción de prácticas ecológicamente sostenibles. Se ponderó, en este estudio, la fase investigativa solamente, cuyos datos se recopilaron mediante entrevistas individuales y se examinaron a la luz del análisis temático reflexivo.

**Resultados:**

Las mujeres identificaron la atención de Enfermería como un apoyo esencial para la organización y la seguridad del entorno laboral. Las percepciones indican que la presencia del personal de enfermería en el día a día de la asociación favoreció el reconocimiento del valor social de su trabajo y el fortalecimiento de la autoestima. Estos hallazgos consolidan la atención de enfermería como una práctica de emprendimiento social, intrínsecamente ligada a la salud ambiental.

**Conclusión:**

Las mujeres recicladoras conciben la atención de Enfermería como promotor de sostenibilidad, rompiendo así con el modelo convencional. Desde la perspectiva del emprendimiento social, la atención se consolida como un fenómeno sistémico y relacional, en el que el personal de enfermería es el eslabón esencial entre la salud humana y la integridad del planeta.

## INTRODUCCIÓN

La enfermería desempeña un papel protagonista en la promoción de acciones ecoorientadas para el logro de los Objetivos de Desarrollo Sostenible^(1,2)^. Los estudios enfatizan la interconexión entre la salud humana y la integridad ecosistémica, con el fin de trascender los enfoques asistencialistas para consolidar conductas sostenibles^(3,5)^. En este contexto, el enfermero asume una función estratégica en la educación ambiental y en la mitigación de riesgos ocupacionales^(6,7)^.

Desde la perspectiva del emprendimiento social, esta atención converge hacia un protagonismo colaborativo junto a las comunidades vulnerables. Al actuar en la confluencia entre la vida y el medio ambiente, el enfermero potencia el saber de los grupos marginados, convirtiendo los desafíos sociales en oportunidades de desarrollo colectivo^(8,9)^. Así, el cuidado se afirma como un fenómeno sistémico y emprendedor, esencial para la dignidad humana y la sostenibilidad de la vida en su dimensión individual y colectiva^(10,11)^.

Sin embargo, persiste una brecha científica sobre la articulación del cuidado con la praxis de las comunidades periféricas en contextos de vulnerabilidad socioambiental, más específicamente en el contexto laboral de las mujeres recicladoras. Estudios previos ratifican la urgencia de traspasar las abstracciones teóricas para investigar el potencial del cuidado en la promoción de la autonomía, la dignidad y la visibilidad de estos grupos tradicionalmente marginados^(6,12)^. Desde esta perspectiva, el enfermero emerge como un agente estratégico, capaz de integrar la preservación de la vida con el desarrollo sostenible local^(13)^.

A partir de lo expuesto, se plantea la siguiente pregunta de investigación: ¿cuáles son las percepciones de las mujeres recicladoras sobre el cuidado de enfermería en la promoción de prácticas ecológicamente sostenibles? Se busca, para ello, comprender las percepciones de las mujeres recicladoras sobre el cuidado de enfermería en la promoción de prácticas ecológicamente sostenibles, desde la perspectiva del emprendimiento social.

## MÉTODO

### Diseño del Estudio

Se trata de una investigación-acción^(14)^ realizada en una asociación de reciclaje en el sur de Brasil, vinculada al proyecto “Emprendimiento Social de Enfermería”. La estructura del informe siguió los criterios de los Criterios Consolidados para el Informe de Investigación Cualitativa (COREQ)^(15)^.

### Escenario del Estudio

La población estuvo compuesta por 32 trabajadores, de los cuales 26 mujeres conformaron la muestra final (sin rechazos). Los criterios de inclusión fueron: ser mujer y haber participado en intervenciones previas sobre prácticas sostenibles (uso de EPI, gestión de residuos y autocuidado). Se excluyó a aquellas que se encontraban de baja laboral durante la recopilación de datos. Las intervenciones se llevaron a cabo semanalmente, según un calendario acordado previamente con las participantes, basadas en metodologías activas sustentadas en el emprendimiento social.

### Recolección y Organización de los Datos

Los datos se recolectaron, entre agosto de 2024 y marzo de 2025, mediante entrevistas individuales, con una duración promedio de 20 minutos, en días, horarios y lugares (espacio privado) previamente acordados con cada una de las participantes. Las entrevistas fueron realizadas por una investigadora con doctorado y experiencia en investigación cualitativa, quien había participado previamente en las intervenciones sistemáticas. Las preguntas que orientaron las entrevistas fueron: Cuénteme sobre su trabajo aquí en la Asociación de Reciclaje. ¿Qué entiende usted por cuidado ecológicamente sostenible? ¿Cuál es la relación entre el cuidado ecológico y su bienestar? ¿Qué actividades considera importantes y que pueden promover el bienestar y las prácticas ecológicamente sostenibles? El cierre de la recopilación se determinó por la saturación teórica, verificada cuando los relatos dejaron de aportar nuevos elementos para la densidad de los códigos y la profundización de los temas emergentes^(16)^.

La reflexividad de la investigadora se ejerció de manera continua, a partir de una inserción previa en el campo, lo que permitió establecer un vínculo de confianza y la participación en la recolección de datos. Se utilizó el diario de campo para separar las impresiones de la investigadora de los hechos narrados, con el fin de garantizar el rigor metodológico y la autocrítica.

Las interpretaciones se presentaron a las participantes en una reunión específica para su validación y la incorporación de elementos, momento que también se grabó en audio.

### Análisis de los Datos

Se utilizó el Análisis Temático *Reflexive* ^(17)^, a partir de las seis fases: familiarización, generación de códigos, búsqueda de temas, revisión, definición y elaboración del informe. El software ATLAS.ti (v.8.0) ayudó en la organización de los datos. Dos investigadoras realizaron la codificación de forma independiente (39 códigos iniciales), que se refinaron de común acuerdo con un tercer investigador, lo que dio como resultado 37 códigos finales organizados en tres temas y nueve subtemas. La codificación independiente por parte de dos investigadoras y el refinamiento por consenso tuvieron como objetivo mitigar interpretaciones puramente subjetivas, asegurando que los núcleos de sentido estuvieran anclados en la realidad empírica. El conjunto de datos de la investigación estará disponible con el autor correspondiente y será accesible previa solicitud razonable.

### Aspectos Éticos

A lo largo del proceso de investigación se observaron las recomendaciones de la Resolución n.º 466/2012 del Consejo Nacional de Salud. El proyecto de investigación fue aprobado por la Comisión Nacional de Ética e Investigación, bajo el dictamen 5.615.579/2022. La participación fue voluntaria, mediante la firma del Término de Consentimiento Libre e Informado y la garantía de poder retirarse en cualquier momento. Para mantener el anonimato, las declaraciones de las participantes se identificaron, a lo largo del texto, con la letra “M”, de Mujer, seguida de un número correspondiente al orden de las declaraciones: M1...M26. Los datos permanecerán bajo la custodia de la investigadora principal durante cinco años.

## RESULTADOS

Las 26 mujeres tenían entre 19 y 64 años, con un promedio de cuatro a ocho hijos. La rutina laboral es de ocho horas diarias, con un ingreso mensual de entre 600,00 y 1.100,00 reales.

El análisis de los datos dio como resultado tres temas centrales, a saber: Cuidado de Enfermería *versus* Cuidado Ecológico; Ambiente integrador y promotor del bienestar; Estrategias potenciadoras del bienestar y del cuidado ecológico. La estructura detallada de temas, subtemas y ejemplos de unidades de registro se resume en la Tabla 1, seguida de la descripción analítica de cada eje temático.

**Tabla 1:** Temas, subtemas y unidades de registro.

Temas centrales	Subtemas	Unidades de registro
Cuidado de enfermería frente a cuidado ecológico	La identidad de la enfermera del medio ambiente. La indisociabilidad entre la salud humana y la salud del planeta. Una visión sistémica e integral de la atención.	““*Somos enfermeras del medio ambiente”; “Todo es cuidado o no cuidamos nada”; “El cuidado ecológico está relacionado con las enfermedades*”.
Un ambiente acogedor que fomenta el bienestar	El espacio de trabajo como refugio y apoyo. La salud como concepto amplio y relacional. El medio ambiente como sujeto y no como objeto.	“*Aquí conversamos y nos reímos”; “La salud está relacionada con el medio ambiente”; “No podemos tratar el medio ambiente como un objeto”; “Mi bienestar influye directamente en el bienestar de los demás”; “La salud está relacionada con la limpieza, el entorno y las relaciones interpersonales*”.
Estrategias para potenciar el bienestar y el cuidado del medio ambiente	Priorización de la salud mental y emocional. Desafíos de la sobrecarga y la rutina agotadora. Sostenibilidad en las relaciones laborales	“*Trabajo mejor cuando consigo relajar la mente y el cuerpo”; “Los días son ajetreados, me acuesto tarde y me levanto temprano”; “El trabajo en equipo mejora mucho nuestro trabajo*”.

### Cuidado de Enfermagem versus Cuidado Ecológico

Las participantes identifican una relación directa entre sus actividades de reciclaje y la preservación de la salud pública. Al autodenominarse “enfermeras del medio ambiente”, estas mujeres le dan un nuevo significado al trabajo manual, considerándolo una acción estratégica para cuidar la vida y la sostenibilidad. Los relatos demuestran que esta percepción eleva la importancia del trabajo de clasificación de residuos, transformándolo en una práctica de protección ambiental que beneficia a la comunidad.

*Nuestro cuidado aquí es muy importante. Cuidamos el medio ambiente, ayudamos a mantener la ciudad limpia y bien cuidada. Somos Enfermeras del Medio Ambiente*. (M1)

*Cuidamos la vida del Planeta, de todo, de todo lo que existe. Todas las cosas forman parte del Planeta y necesitan ser cuidadas. ¿Y quién debe cuidar? Todos nosotros. Aquí siempre recordamos y decimos que somos Enfermeras del Medio Ambiente.* (M5)

El análisis de los discursos revela la indisociabilidad entre las dimensiones asistencial y ecológica del cuidado. Para las participantes, el ejercicio de la enfermería y el trabajo de reciclaje convergen en el objetivo común de garantizar condiciones ambientales favorables para la vida. Esta integración valida la comprensión de que la salud humana depende de la integridad de los ecosistemas, lo que motiva a las trabajadoras a reconocer su papel en el mantenimiento del equilibrio planetario y en la prevención de problemas de salud.

*No se puede separar una cosa de la otra. Todo es cuidado o no cuidamos. Un enfermero cuida al paciente, pero el paciente depende del aire que respira, depende del clima, del agua, de la limpieza, del medio ambiente.*(M10)

*Necesitamos el cuidado y, por lo tanto, todas las cosas necesitan cuidado. Y, si no cuidamos, ¿qué pasa? ¡Más enfermedades! Lo que hacemos aquí es cuidar la salud del medio ambiente.* (M14)

Las narrativas reiteran que el proceso de salud-enfermedad está influenciado por las condiciones climáticas, sanitarias y por la preservación de los recursos naturales. Al señalar que la asistencia sanitaria depende de la calidad del aire y del agua, las mujeres demuestran conciencia sobre los determinantes socioambientales. Esta percepción indica que el cuidado debe abarcar el medio ambiente como elemento central para el mantenimiento de la vida, ampliando la visión sobre las prácticas terapéuticas tradicionales.

*La salud está relacionada con el aire que respiramos, el agua que tomamos. No se puede separar nuestro trabajo del cuidado de la salud.* (M19)

Los datos revelan que las recicladoras perciben el cuidado de enfermería como una práctica social emprendedora. La identificación de las trabajadoras con la figura de la “enfermera del medio ambiente” pone de manifiesto que la promoción de prácticas sostenibles se entiende como una extensión del cuidado de la salud, donde la clasificación de residuos se convierte en la herramienta operativa para garantizar la dignidad humana y la vitalidad del ecosistema.

### Ambiente Integrador y Promotor del Bienestar

Para las participantes, el bienestar se configura como un fenómeno vinculado a la calidad del ambiente y al establecimiento de interacciones interpersonales de apoyo mutuo. Desde esta perspectiva, el escenario laboral en la asociación trasciende la función productiva para convertirse en un espacio de apoyo emocional y seguridad, actuando como un factor de protección que ayuda a enfrentar los desafíos cotidianos. A pesar de las dificultades estructurales, las trabajadoras valoran la convivencia entre colegas como un elemento central para el mantenimiento de la salud mental y la satisfacción en el trabajo.

*Me siento bien trabajando aquí en l*a Asociación.

*Claro que es un trabajo agotador y poco valorado. Pero me siento muy bien aquí, porque en casa no tengo espacio ni ambiente. Cuando venimos al trabajo, tenemos a las compañeras con quienes conversar, cómo decirlo.* (M3)

Los datos revelan que el escenario laboral se caracteriza como un territorio de intercambios subjetivos, donde el diálogo y la acogida entre las mujeres minimizan el desgaste de la rutina agotadora. Esta percepción amplía el concepto de salud más allá de la ausencia de enfermedades, integrándolo a la calidad de las relaciones y a la organización del entorno. Las participantes asocian la limpieza del lugar de trabajo y la armonía del grupo como componentes vitales que impactan directamente en el ánimo y la capacidad productiva.

*En el trabajo siempre nos sentimos bien y apoyadas. Siempre estamos conversando y riendo. Entonces, este es el sentido del cuidado, no hay palabras*. (M5)

*La salud está relacionada con la limpieza, el entorno y las relaciones interpersonales. Trabajar en un lugar limpio y con las compañeras unidas marca toda la diferencia para que podamos aguantar el día.* (M12)

Las narrativas indican que la preservación del ambiente de trabajo no se ve solo como una norma técnica, sino como una condición para la dignidad de las trabajadoras. Al tratar el entorno como un elemento que influye en el estado emocional y físico, las mujeres refuerzan que el mantenimiento de un espacio organizado y acogedor es una estrategia de cuidado colectivo. Este cuidado por el ambiente se refleja en la cooperación diaria, donde el cuidado de las demás y del espacio se fusionan para garantizar la continuidad de las actividades.

*No podemos tratar el ambiente como un objeto... Cuidamos el galpón porque el galpón nos cuida a nosotras. Si todo está sucio y desordenado, nos enfermamos juntas.* (M18)

Las declaraciones demuestran que las prácticas de cuidado, como el descanso y la escucha activa, son fundamentales para sostener la resiliencia en el entorno del reciclaje. Para las participantes, el bienestar colectivo es fruto de una cultura de cuidado que armoniza las necesidades individuales con la organización del espacio compartido. Al reconocer la asociación como un espacio de refugio y apoyo, las mujeres validan la premisa del emprendimiento social de que la transformación de la realidad ambiental depende de una base relacional sólida. Así, la promoción de prácticas sostenibles se potencia cuando el Enfermero actúa como facilitador de un ambiente que integra la salud física, emocional y social.

### Estrategias Potenciadoras del Bienestar y del Cuidado Ecológico

Las participantes identifican la salud mental, especialmente el descanso, como estrategias fundamentales para el mantenimiento de la productividad y la calidad de vida. Los relatos demuestran que, ante una rutina física agotadora, la capacidad de relajar la mente y el cuerpo se percibe como una condición para el buen desempeño de las actividades laborales. Para las mujeres, el cuidado de enfermería se manifiesta en el estímulo de pausas restauradoras y en orientaciones que ayudan a equilibrar las exigencias del trabajo y las necesidades personales, como se indica a continuación:

*Lo principal es la salud mental y emocional, no tanto la física, que vamos llevando. Cansarse es algo que nos pasa a todos, pero creo que lo principal es la cabeza, la mente. Sentirme bien conmigo misma y con los demás. Esto tiene que ver con el ambiente, con el clima, con el aire que respiro*. (M13)

*Creo que debería haber actividades enfocadas en la mente, algo que ayude a relajar la mente y el cuerpo. A veces hay muchas cosas en la cabeza, ¿no? El ambiente se vuelve pesado, agotador, se cometen errores.* (M16)

Los datos revelan que la cooperación y el trabajo en equipo actúan como mecanismos de sostenibilidad en las relaciones laborales. La unión entre las recicladoras se señala como una estrategia que facilita el flujo de las actividades y reduce el impacto de la sobrecarga física. Esta percepción pone de manifiesto que el cuidado ecológico, para ser efectivo, depende de una estructura de apoyo colectivo en la que la ayuda mutua entre las compañeras optimiza la clasificación de los materiales y promueve un ambiente más armonioso, tal como se expresa a continuación:

*El trabajo en equipo mejora mucho nuestro trabajo. Cuando una ayuda a la otra, la carga se vuelve más ligera y logramos separar todo correctamente.* (M20)

*Nos ayudamos mucho aquí. Si una está cansada, la otra le echa una mano. Eso hace que el día pase mejor y cuidamos mejor las cosas.* (M24)

Las declaraciones indican que la adopción de prácticas ecológicamente sostenibles, como el uso correcto de los equipos y la separación rigurosa de los residuos, está condicionada al bienestar emocional de las trabajadoras. Al asociar el estado de ánimo con la calidad de la ejecución de las tareas, las mujeres refuerzan que la eficiencia en el reciclaje no es solo técnica, sino que depende de condiciones motivacionales y relacionales. Esta comprensión sugiere que el enfermero, al promover la salud mental, fortalece indirectamente la preservación del medio ambiente.

*Para cuidar el medio ambiente, tenemos que sentirnos bien con nosotras mismas. Si estoy feliz y descansada, cuido mucho mejor cada material que pasa por mis manos*. (M21)

En resumen, los resultados demuestran que la promoción de prácticas ecológicamente sostenibles se produce a través de estrategias de cuidado que valorizan la dimensión humana y colectiva del trabajo. Desde la perspectiva del emprendimiento social, los resultados evidencian que el personal de enfermería potencia la sostenibilidad planetaria al intervenir en la salud mental y en la organización del trabajo de las recicladoras. Así, el fortalecimiento de la autonomía y el bienestar emocional de estas mujeres se consolida como la base operativa para el mantenimiento de la vida y el equilibrio ambiental.

## DISCUSIÓN

Los resultados revelan que, en la percepción de las participantes, el cuidado de enfermería y el cuidado ecológico son indisociables, componiendo una red de interdependencia vital. Al utilizar la metáfora simbólica de “Enfermeras del Medio Ambiente”, las recicladoras validan el potencial del cuidado más allá del fenómeno de la enfermedad y correlacionan la clasificación de residuos con la promoción de la salud colectiva. Este hallazgo converge con la literatura contemporánea que posiciona a la enfermería como categoría central en la defensa de la salud planetaria, rescatando el legado histórico de la profesión sobre la salubridad ambiental como principio de la curación^(18,20)^.

El ambiente de la asociación de reciclaje se identificó como un determinante crítico de la salud, al figurar como un territorio de apoyo emocional que trasciende la función productiva. El bienestar, en este contexto, es descrito por las mujeres como un fenómeno relacional, donde la limpieza del entorno y la armonía de los vínculos interpersonales actúan como factores de protección contra el desgaste de la rutina extenuante.

Los estudios sobre salud ocupacional refuerzan que el equilibrio en los entornos laborales es un sistema dinámico, al enfatizar que la calidad de las relaciones y la organización del espacio son fundamentales para la dignidad y la salud mental del trabajador^(21,22)^.

La articulación entre la práctica de las recicladoras y la atención de enfermería demuestra que el impacto de la gestión de residuos en la mitigación de los daños ambientales es un compromiso ético compartido. La percepción de las mujeres de que “el paciente depende del aire y del medio ambiente” ratifica la urgencia de superar el modelo biomédico tradicional, integrando los determinantes socioambientales en la práctica asistencial cotidiana. Las investigaciones refuerzan este pensamiento al demostrar que esta ampliación del ámbito profesional institucionaliza la sostenibilidad como eje estructurante de la promoción de la salud global^(21,24)^.

Desde la perspectiva del emprendimiento social, el protagonismo de las recicladoras revela que el cuidado puede ser una fuerza transformadora y generadora de autonomía en comunidades vulnerables. Al convertir los desafíos sociales en oportunidades de desarrollo colectivo, las participantes demuestran que el cuidado emprendedor de enfermería no es solo una herramienta accesoria, sino una dimensión que confiere un significado tangible al trabajo comunitario. Esta lógica permite generar soluciones creativas que armonizan la eficacia del cuidado con la responsabilidad ambiental, elevando la visibilidad de grupos tradicionalmente marginados^(25,29)^.

La convergencia entre el saber de las recicladoras y el cuidado de enfermería demuestra que el acto de cuidar está directamente vinculado a la preservación del medio ambiente y a la salud humana. Los hallazgos indican que el cuidado, cuando se aplica al contexto del reciclaje, promueve la protección de la salud y la mejora de las condiciones de vida. El estudio corrobora este pensamiento al reforzar el papel del enfermero en la gestión de riesgos y en la valorización del trabajo sostenible, con el fin de salvaguardar la perennidad de la vida en su multidimensionalidad^(30)^.

Desde la perspectiva del emprendimiento social, el protagonismo de las recicladoras revela que el cuidado de enfermería actúa como herramienta impulsora de la autonomía al integrar orientaciones de salud en la rutina productiva^(11,12)^. La conversión de los desafíos sociales en oportunidades de desarrollo se evidencia en la capacidad de las participantes para autogestionar los riesgos y valorar el trabajo colectivo, lo que confiere un sentido práctico y tangible a la actuación de la enfermería en la comunidad.

Las limitaciones de este estudio se relacionan con la especificidad del corte muestral, compuesto por mujeres pertenecientes a una única asociación de recicladoras, lo que circunscribe los hallazgos a ese contexto sociolaboral particular. Debido a su naturaleza cualitativa, los resultados no buscan la generalización, sino la profundidad analítica de experiencias y motivaciones que están influenciadas por dinámicas de género y condiciones de trabajo singulares.

Las contribuciones de este estudio al avance de la ciencia de la enfermería están asociadas a la urgencia de integrar la dimensión ecológica como principio básico de la salud pública. Los resultados ofrecen fundamentos para la inducción y el fortalecimiento de políticas públicas que estimulen la corresponsabilidad en el manejo ético de los residuos sólidos y en la valorización del enfermero como agente estratégico en la intersección entre la vida humana y el equilibrio planetario.

## CONCLUSIÓN

Las percepciones de las recicladoras revelan que la atención de enfermería, al fortalecer la autonomía de las mujeres recicladoras, promueve la seguridad laboral, la organización del entorno de trabajo y el apoyo emocional. La presencia del enfermero en el día a día de la asociación actúa como una intervención de vigilancia en salud, convirtiendo la clasificación de materiales en una práctica consciente de preservación ambiental. El estudio evidencia que la integración del saber de las mujeres con las orientaciones técnicas de salud potencia la adopción de prácticas sostenibles y la valorización de su identidad profesional.

Se concluye que la atención de enfermería, desde la perspectiva del emprendimiento social, trasciende el modelo clínico al intervenir directamente en los determinantes socioambientales y en la dignidad laboral. Por último, se sugiere que las futuras investigaciones sobre recicladores contemplen otras asociaciones y también a los trabajadores del sexo masculino, con el fin de asegurar una mayor representatividad y diversidad, a partir de las particularidades de cada grupo, como la organización, las condiciones de trabajo, las dinámicas internas y otras.

## Data Availability

El conjunto de datos que respaldan las conclusiones de este estudio está disponible previa solicitud al autorcorrespondiente.
